# Food and Beverage Options at Highway Rest Areas in North Carolina: A Mixed-Methods Audit and Geospatial Approach

**DOI:** 10.5888/pcd16.190129

**Published:** 2019-10-17

**Authors:** Jared T. McGuirt, Grace Huebner, Rachel Ward, Stephanie B. Jilcott Pitts

**Affiliations:** 1 Department of Nutrition, University of North Carolina at Greensboro, Greensboro, North Carolina; 2 Department of Community and Behavioral Health, College of Public Health, East Tennessee State University, Johnson City, Tennessee; 3 Department of Public Health, East Carolina University, Greenville, North Carolina

## Abstract

**Introduction:**

Each year, millions of people purchase food at highway rest areas. Rest areas are potential sites for health promotion because they are operated by the public sector; they are frequently visited by professional truck drivers, who have a disproportionate burden of chronic disease; and they are easily accessible. To our knowledge, no research has systematically examined the healthfulness of food offerings at rest areas. The objective of this study was to determine the accessibility and healthfulness of food and beverages offered at highway rest areas in North Carolina using a mixed-methods audit and geospatial approach.

**Methods:**

We conducted a cross-sectional audit of all rest areas offering foods and beverages in North Carolina (N = 30) in summer 2018. We used the Nutrition Environment Measures Survey–Vending (NEMS–V) to record the 1) type, price, and size of all foods and beverages and 2) healthfulness of items offered (based on NEMS–V categorization). Two researchers independently double coded NEMS–V data. We used geospatial analysis to examine proximity of rest areas to food stores. We analyzed data by using univariate and bivariate analysis.

**Results:**

The mean number of vending machines per site was 8.0 (range, 2–12, standard deviation, 2.8). The healthfulness of offerings varied across sites. Most food items (88.1%; 2,922 of 3,315) and beverage items (63.7%; 1,567 of 2,459) were classified as least healthful. Cold beverage machines had a greater percentage of healthful items (38.2%; 778 of 2,036) than snack machines (11.4%; 374 of 3,270) (*P* < .001), mainly because of water and diet soda in beverage machines.

**Conclusion:**

Policy changes are needed to increase the number and presentation of healthful food options at highway rest areas. Policy changes could provide travelers with more healthful options conveniently located along their travel route.

**Summary**

**What is already known on this topic?**

Millions of people purchase food and beverages at highway rest areas. These sites receive little attention in food environment research, despite their potential for health promotion, particularly among long-distance truck drivers, who have a disproportionate burden of chronic disease.

**What is added by this report?**

We found that most food and beverages at highway rest areas in North Carolina were unhealthful and that the healthfulness of items varied across sites.

**What are the implications for public health practice?**

Policy changes are needed to increase healthful food options and presentation at highway rest areas, which would provide travelers with more healthful options conveniently located along their travel route.

## Introduction

Consumers going about activities of daily life increasingly prioritize convenience and accessibility when choosing ready-to-eat food products (1,2). In 2012, 86% of Americans reported regularly eating or drinking in the car, often as a result of time constraints and mobile lifestyles (3). The longer on the road, the more likely drivers are to want a quick and easy stop for food (4). Thus, drivers, particularly long-distance truck drivers, who have a disproportionate burden of chronic disease, are a strategic target for encouraging healthful food consumption (5,6).

One commonly used source of convenient food purchases, particularly among truckers, is highway rest areas. The United States has more than 1,800 rest areas (7). These sites receive little attention in food environment research, even though each year millions of people in the United States purchase snacks and beverages at these rest areas (24 million people per year in North Carolina alone) (8).

Rest areas are potentially useful sites for health promotion because they are owned and operated by the public sector (typically state departments of transportation); they are frequently visited; and they are easily accessible to truckers and long-distance travelers, including families looking for snacks and beverages for their children. Rest areas are often isolated, because private businesses, such as fast food restaurants, cannot locate in the immediate proximity of rest areas (9). Because they are located directly off exits and drivers do not need to navigate through intersections, rest areas are easier to access than other highway food sources.

To our knowledge, no research has systematically examined the food environment at highway rest areas. The objective of this study was to determine the accessibility and healthfulness of food and beverages offered at rest areas in North Carolina by using a mixed-methods audit and geospatial analysis.

## Methods

We conducted a cross-sectional audit of all toll-free vending-only highway rest areas offering foods and beverages in North Carolina in June and July 2018. Research staff members identified all rest areas in the state by using the North Carolina Department of Transportation website (10) and filtered data to identify locations offering food and beverages for sale. Most (69.8%, 30 of 43) rest areas had food and beverages for sale. Research staff members then traveled to each rest area offering food and beverages to conduct a food environment assessment.

The research team used the Nutrition Environment Measures Survey–Vending (NEMS–V) to record the availability of food choices in vending machines. The team recorded the following: vending machine location (site of rest area and location of machine within site); the type, price, and size (in ounces) of foods and beverages; NEMS–V defined type of vending machine (cold beverage, hot beverage, snack, combination [refrigerated and nonrefrigerated beverage and snack], and ice cream); item location within the machine; and healthfulness of items offered (11). The team also rated the cleanliness of each vending machine as acceptable or not acceptable and noted whether advertising of any item was posted.

The research team rated the healthfulness of each food and beverage item and the overall healthfulness of vending machines by using the NEMS–V food coding summary (11). The NEMS–V uses nutrition criteria of the Health and Sustainability Guidelines for Federal Concessions and Vending Operations (12). These standards are based on multiple factors, including calories, sodium content, and percentage of calories from fat. We labeled each food (excluding gums and mints, because they have negligible nutritional value) and beverage item as green (healthiest choice), yellow (healthy choice), or red (not as healthy of a choice) and then summed the total number of each color in each machine. We classified each machine into the following NEMS–V “medal” categories of healthfulness (11): no medal (<30% of food items and <55% of beverage items were yellow or green), bronze (at least 30% of food items or 55% of beverage items were yellow or green), silver (at least 40% of food items or 65% of beverage items were yellow or green), or gold (at least 50% of food items or 75% of beverage items were yellow or green).

Two researchers independently completed all NEMS–V assessments. After completion of the audits, the research team met, reached consensus on all items, and developed a final data set for analysis.

To examine the spatial context of rest area sites and the potential influence of contextual characteristics (eg, competition from nearby food stores, rural vs urban location) on the healthfulness of items offered, the research team used ArcGIS version 10.4.1 (Esri). We identified limited-service restaurants (North American Industry Classification System [NAICS]-722513) and convenience stores (NAICS-445120) by accessing the Reference USA commercial business database (7). Limited-service restaurants are primarily establishments where patrons typically order or select items and pay before eating; food and beverages may be consumed onsite, taken out, or delivered. We batch-coded the location of rest areas, limited-service restaurants, and convenience stores to the highest level of accuracy with the Google Maps application programming interface through the BatchGeo website (13). Using Esri’s ArcGIS Spatial Analyst, we generated buffer service areas along road networks from the rest area sites and joined these to food venue point layers to obtain the number of limited-service restaurants and convenience stores within defined areas. To account for differences in travel patterns based on these various highway configurations, we created 0.5-mile, 1-mile, 2-mile, and 5-mile road network buffers for rest areas on traditional grid highways (not limited access, at-grade intersections [ie, where roads connect at a common elevation]), and 2-mile, 5-mile, 10-mile, and 20-mile road network buffers for rest areas only on limited-access highways (with interchanges and ramps). We determined whether rest areas were located in rural areas or urban areas by using US Census Bureau urbanized area shapefiles (14).

We generated univariate statistics for frequencies or percentages of vending machine types overall and by site; the average number of items per machine overall and by site; the most common foods and beverages; the average, range, and variability of package sizes and prices across items; the frequency and percentage of each healthfulness color code overall and by site; and the frequency and percentage of vending machine medal level overall and by site. We also generated frequencies for the number of limited-service restaurants and convenience stores within various distances of the rest areas. At the vending machine level, we used the Fisher exact test to measure differences in frequency of each healthfulness color code by machine type. At the site level, we used χ^2^ tests to examine 1) the number of machines that received a medal (bronze, silver, gold) and the number that did not and 2) the overall number of green or yellow items and the overall number of red items. We conducted the χ^2^ analysis by whether the rest area was located in a rural or urban area and whether it was located on an interstate or not. We used R Studio version 3.43 (RStudio: Integrated Development for RStudio, Inc) for analysis.

## Results

All machines were working at almost all sites (28 of 30) (Table 1). We found 24 rural rest areas and 6 urban rest areas; most sites were located along interstate highways (23 of 30), and the remaining sites were located on primary state highways (7 of 30) (Figure). Of the 7 primary state highways, 1 was a limited-access highway, 3 were traditional grid highways, and 3 had characteristics of both. Nearly half of the rest areas (14 of 30) had no limited-service restaurants within 5 miles, and 9 of 30 rest areas had no convenience stores within 5 miles. The mean number of stores within a 5-mile buffer of a limited-access highway was 8.7 for limited-service restaurants and 7.0 for convenience stores (Table 2).

**Table 1:** Characteristics of Highway Rest Areas in North Carolina (n = 30), Summer 2018^a^

Variable	Result
**All vending machines were working**	93.3% (28 of 30)
**Located in rural area**	80.0% (24 of 30)
**Type of highway**	
Interstate highway	76.7% (23 of 30)
Primary state highway	23.3% (7 of 30)
Limited-access highways (with interchanges and exit ramps)	14.3% (1 of 7)
Traditional grid (not limited-access, at-grade intersections [ie, roads that connect at common elevation])	42.9% (3 of 7)
Both limited access and traditional highways (junction)	42.9% (3 of 7)
**No. of machines per site, mean (SD)**	
Total	8.0 (2.8)
Cold beverage	4.1 (1.5)
Snack machines	2.9 (1.2)
Hot beverage	0.6 (0.5)
Combination	0.2 (0.3)
Ice cream	0.1 (0.4)
**Mean percentage (SD) of individual items by healthfulness category^b^ across sites**	
Green or yellow	22.1 (4.2)
Red	77.8 (4.2)
**Site-level healthfulness awards for machines^c^**	
No machines were given a medal	40.0% (12 of 30)
Bronze	33.3% (10 of 30)
Silver	10.0% (3 of 30)
Gold	36.7% (11 of 30)
>1 Medal	20.0% (6 of 30)
**Urban versus rural**	
Green and yellow versus red	χ^2^ = 0.6; *P* = .43
No medal versus medal (bronze, silver, or gold)	χ^2^ = 1.1; *P* = .30
**Interstate versus noninterstate**	
Green and yellow versus red	χ^2^ = 0.8; *P* = .37
No medal versus medal (bronze, silver, gold)	χ^2^ = 0.2; *P* = .63

a Data source: North Carolina Department of Transportation (10). Of 43 highway rest areas in the state, 30 offered foods and beverages for sale in vending machines in June and July 2018.

b Items were coded according to healthfulness of items. The NEMS–V uses nutrition criteria of the Health and Sustainability Guidelines for Federal Concessions and Vending Operations (12). These standards are based on multiple factors, including calories, sodium content, and percentage of calories from fat. Green, healthiest choice; yellow, healthy choice; red, not as healthy a choice.

c Award categories: no medal, <30% of food and <55% of beverage items were yellow or green; bronze medal, at least 30% of food or 55% of beverage items were yellow or green; silver medal, at least 40% of food or 65% of beverage items were yellow or green; gold medal, at least 50% food or 75% beverage items were yellow or green.

**Figure:**
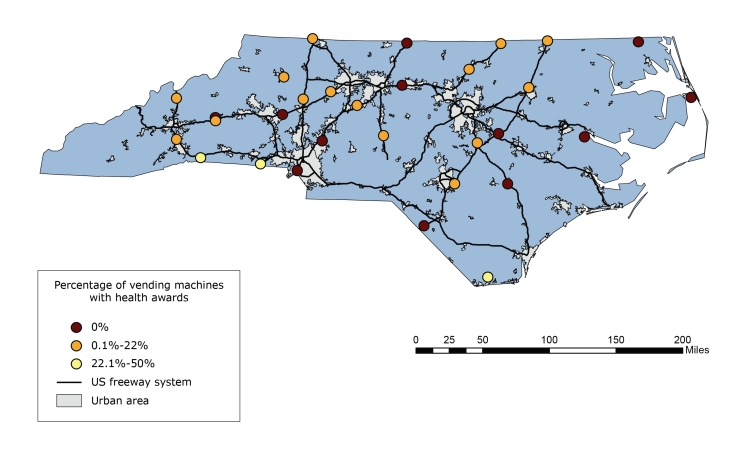
Map of highway rest area locations serving food and/or beverages in vending machines, North Carolina (N = 30). Each location was categorized as bronze, silver, gold, or no award, on the basis of criteria established by the Nutrition Environment Measures Survey–Vending (11). US Census Bureau–designated urban areas and the US freeway road network are also indicated.

**Table 2:** Proximity of Highway Rest Areas on Different Highway Types (Limited-Access Highway vs Not a Limited-Access Highway) to Limited-Service Restaurants or Convenience Stores, North Carolina, Summer 2018^a^

Road Network Buffer^b^	No. of Stores Within Buffer Distance	
	Limited-Access Highway^c^ (n = 24), Mean (IQR)	Not a Limited-Access Highway (n = 6), Mean (IQR)
**Limited-service restaurant**		
0.5 mile	—d	0.43 (0–0)
1 mile	—d	0.6 (0–0.8)
2 miles	1.0 (0–0)	3.3 (0–5.3)
5 miles	8.7 (0–11.5)	7.8 (0–13)
10 miles	32.2 (5–39.8)	—d
20 miles	98.7 (29.3–87.3)	—d
**Convenience store**		
0.5 mile	—d	0.1 (0–0)
1 mile	—d	0.8 (0–1.8)
2 mile	0.5 (0–0.3)	2.5 (0.3–4.5)
5 mile	7.0 (0–12.8)	7.2 (1.0–9.0)
10 mile	29.3 (7.0–40.3)	—d
20 mile	83.5 (40.3–105.5)	—d

a Data source: North Carolina Department of Transportation (10). Of 43 highway rest areas in the state, 30 offered foods and beverages for sale in vending machines in June and July 2018.

b Buffer area generated on and following road networks.

c Characterized by interchanges and exit ramps.

d Not measured because measure not appropriate for highway type.

The mean number of machines per site was 8.0 (range, 2–11; standard deviation [SD], 2.8). Cold beverage machines were the most common machines across the sites, with a mean of 4.1 (SD, 1.5) machines per location, followed by snack machines (mean, 2.9; SD, 1.2), hot beverage (mean, 0.6; SD, 0.5), combination (mean, 0.2; SD, 0.3), and ice cream (mean, 0.1; SD, 0.4) (Table 1). For healthfulness awards, 12 sites had no machines with an award, 10 sites had a machine with a bronze award, 3 sites had a machine with a silver award, and 11 sites had a machine with a gold award. Six sites had more than 1 machine that received a medal. The highest proportion of machines that received a medal at any site was 1 of 2 machines.

In the comparison of urban and rural locations, we found no significant difference between the number of green or yellow items and the number of red items (χ^2^ = 0.6; *P* = .43) or between the number of machines with no medal and the number of machines with a medal (χ^2^ = 1.1; *P* = .30). In the comparison of interstate locations and noninterstate locations, we found no significant difference between the number of green or yellow and the number of red items (χ^2^ = 0.8; *P* = .37) or between the number of machines with no medals and the number of machines with medals (χ^2^ = 0.2; *P* = .63) (Table 1).

### Vending machines

We found 241 vending machines across all sites (Table 3); nearly all (n = 239) were in working order. We found no instances of advertising of healthful or unhealthful food or beverage items. All vending machines were considered acceptably clean. We found a mean 24.0 (SD, 14.3) items per machine.

**Table 3:** Characteristics of Vending Machines (N = 241) at Highway Rest Areas in North Carolina, by Type of Vending Machine, Summer 2018^a^

Characteristic	Type of Vending Machine					
	All Vending Machines (N = 241)	Cold Beverage (n = 125)	Snack (n = 88)	Hot Beverage (n = 19)	Combination^b^ (n = 5)	Ice Cream (n = 4)
**No. of items per vending machine, mean (SD)**						
Overall	24.0 (14.3)	16.3 (12.9)	37.2 (5.3)	19.1 (7.6)	10.8 (3.9)	12.8 (7.9)
Green items^c^	2.0 (2.8)	2.5 (3.4)	1.0 (1.1)	3.0 (1.7)	1.0 (1.1)	0
Yellow items^c^	3.4 (2.8)	3.8 (3.4)	3.2 (2.0)	2.7 (1.6)	3.8 (1.7)	0.2 (0.4)
Red items^c^	18.6 (13.2)	10.1 (9.0)	32.9 (5.5)	13.4 (5.9)	6.0 (1.9)	12.5 (8.0)
**Award level^d^**						
No medal	89.2% (215 of 241)	82.4% (103 of 125)	98.9% (87 of 88)	94.7% (18 of 19)	60.0% (3 of 5)	100% (4 of 4)
Bronze medal	4.6% (11 of 241)	7.2% (9 of 125)	1.1% (1 of 88)	0	20.0% (1 of 5)	0
Silver medal	1.7% (4 of 241)	2.4% (3 of 125)	0	0	20.0% (1 of 5)	0
Gold medal	4.6% (11 of 241)	8.0% (10 of 125)	0	5.3% (1 of 19)	0% (0 of 5)	0

a Data source: North Carolina Department of Transportation (10). Of 43 highway rest areas in the state, 30 offered foods and beverages for sale in vending machines in June and July 2018.

b Refrigerated and nonrefrigerated beverage and snack.

c Items were coded according to healthfulness of items. The NEMS–V uses nutrition criteria of the Health and Sustainability Guidelines for Federal Concessions and Vending Operations (12). These standards are based on multiple factors, including calories, sodium content, and percentage of calories from fat. Green, healthiest choice; yellow, healthy choice; red, not as healthy a choice.

d Award categories: no medal, <30% of food and <55% of beverage items were yellow or green; bronze medal, at least 30% of food or 55% of beverage items were yellow or green; silver medal, at least 40% of food or 65% of beverage items were yellow or green; gold medal, at least 50% food or 75% beverage items were yellow or green.

Most items (61.8%; 1,258 of 2,036) in cold beverage machines were categorized as red; 38.2% (778 of 2,036) were categorized as yellow or green. Of 125 cold beverage machines, most (82.4%; n = 103) received no award; 8.0% (n = 10) were categorized as gold, 7.2% (n = 9) as bronze, and 2.4% (n = 3) as silver. Among 88 snack machines, 88.6% (2,896 of 3,270) of items were categorized as red; 11.4% (374 of 3,270) were categorized yellow or green. Most (98.9%; n = 87) snack machines received no award. Among the 5 combination machines, 55.6% (30 of 54) of items were categorized as red and 44.4% (24 of 54) as yellow or green. Among 19 hot beverage machines, 70.2% (255 of 363) of items were categorized as red (primarily because of coffee drinks with both added sugars and creamers), and 29.8% (108 of 363) of items were categorized as yellow or green (primarily because of plain coffee, sugar or creamer added [but not both], and hot water). Among the 4 ice cream machines, 98.0% (50 of 51) of items were categorized as red and none as green.

Cold beverage machines were significantly more likely than snack machines to receive a bronze, silver, or gold medal (*P* < .001), but we found no significant differences when comparing cold beverage machines with hot beverage machines (*P* = .21), combination machines (*P* = .23), or ice cream machines (*P* = .58).

### Food and beverage items

We found 5,744 items across all sites: 3,315 food items and 2,459 beverage items. The mean portion size of food items was 1.9 oz (range, 0.5–15.0 oz; SD, 1.1 oz), and the mean price was $1.19 (range, $0.45–$3.50; SD, $0.39). Mean beverage portion size was 17.2 oz (range, 1.93 oz [energy drink] to 24.0 oz; SD, 4.0 oz), and mean price was $1.63 (range, $0.75–$3.25; SD, $0.51). Overall, most (88.1%; n = 2,922) food items were categorized as red; 9.0% (n = 300) were categorized as yellow and 2.8% (n = 93) as green. Most 63.7% (n = 1,567) beverages were categorized as red; 21.3% (n = 523) were categorized as yellow; and 15.0% (n = 369) as green. The most common “green” beverage was plain bottled water (92.7%; 342 of 369), and the most common “yellow” beverage was Diet Pepsi (15.7%; 82 of 523). The most common “green” food was salted peanuts (n = 39), followed by trail mix (n = 30, all brands) and Goldfish crackers (n = 25). The 5 most common “red” foods were Skittles (n = 129; all flavors), Lays potato chips (n = 109; all flavors), Doritos (n = 81; all flavors), Snickers (n = 74), and Peanut M&Ms (n = 61).

## Discussion

To our knowledge, this is the first study to describe the healthfulness of food and beverages offered at highway rest areas. Our findings confirm reports that public area vending machines offer few healthful options (15,16). Although most items and machines were classified as less healthful, we found generally healthful options, such as water and nuts, at every site. More healthful options could be promoted to travelers to encourage their selection, including product placement for greater visibility, promotional signage, and price discounts. We also found that machines often had either mostly less healthful options or mostly healthful options. Further research is needed to understand which would result in better choices: combining unhealthful and healthful items or isolating healthful items in their own machines. One study found that the most intrusive vending machine intervention (highest restriction on energy-dense options) led to lower-calorie choices than did less intrusive interventions, such as calorie labeling, increasing low-calorie choices, and increasing the price of high-calorie choices (17). That study, however, was conducted in a research laboratory and not a real-world setting, and previous research found intrusive interventions are less likely to be widely accepted by the public (18,19).

We found some variability across sites of the healthfulness of items but no significant differences in the healthfulness of items by urban–rural designation or proximity to the interstate. Factors driving the variability in the healthfulness of items offered are unclear, particularly given that rest areas in North Carolina are operated by a single public entity and serviced by vending contracts with the state department of transportation. Future research should examine these influential factors. Previous vending trade reports suggest spatial variability in the success of certain products (20).

Items that could be deemed as more nutrient-dense alternatives may have been categorized as unhealthful in our study. For example, we categorized vegetable chips and straws as red items because of their percentage of saturated fat. Thus, the perceived accessibility to more healthful options could be deflated by the strict categorization used in NEMS–V scoring. Small changes from less nutrient-dense items to more nutrient-dense items, even if not a leap in healthfulness, can be meaningful in achieving a better nutritional status and could be a useful strategy to promote more healthful purchases.

Making more healthful options more available or highlighting more healthful items could be an effective state-level policy to improve health behavior. More refrigerated machines with fresh produce and other more healthful foods may be needed to improve the healthfulness of vending items. Although increasing availability of bottled water could create more healthful vending options, the promotion, enhancement, or installation of hydration stations (ie, water bottle refilling stations) could be a better approach because of the negative environmental impact of plastic bottles. Another approach to increase the availability of more healthful foods could be direct-to-consumer operations, such as produce stands and farmers markets run by local farmers. This approach was adopted in some areas to promote local agriculture (21). More healthful options could also be promoted in the same way other non-diet–related health behavior initiatives have been promoted at highway rest areas. Several US states have established “safe phone zones” in rest areas, where travelers are offered a safe place to use their cell phones (22). Such interventions could target both commuting and long-distance motorists.

Although research on rest area usage is limited, a report in New England found that vending machines are a top amenity for truck drivers (23), who are more likely than other motorists to use rest areas as a food source because the size of their vehicle limits their choices (24). It is important for truckers to have safe areas to rest and opportunities to eat more nutritious foods; these factors may help truckers to be alert on the road and prevent accidents. In 2017, 12% of traffic fatalities involved a large truck or bus (25). The National Academy of Sciences, Engineering, and Medicine suggested the need to further examine causes of commercial driver fatigue, some which could be diet-related (26). Truckers are a key part of our national economy and keeping them healthy and strong is of national public health and economic interest.

Policies were recently enacted or proposed to improve the offering of more healthful foods in vending machines to combat the obesity epidemic (27). Glendale, California, voted to replace all chips and sodas with fruits, vegetables, and nuts in vending machines on city property (28). Maryland tried, albeit unsuccessfully, to require that at least 75% of packaged food and beverage options offered in a food and beverage vending machine located on property owned or managed by the state to be healthful options (29). In addition, 3 federal agencies released regulations or recommendations on healthful vending. The US Department of Agriculture’s Smart Snacks in School regulation requires that foods served in vending machines at schools meet nutrition standards (30). The US Department of Health and Human Services developed Food Service Guidelines for Federal Facilities, which sets goals to ensure that healthful foods and beverages are encouraged at all federal facilities (31). The Centers for Disease Control and Prevention developed a guidebook, *Healthy Vending Machine Initiatives in State Facilities* (32). Each policy is an example of a large-scale public policy to inform future initiatives to increase healthful options in rest area vending machines.

This study has limitations. We collected data in only 1 state, and the data may not be representative of items offered in rest areas elsewhere in the United States. Future research should examine the healthfulness of items offered in a larger geographic area. North Carolina offers vending options only along toll-free highways, whereas some states have extensive toll road systems with commercialized areas that have multiple options for dining. Thus, future research should examine the food offerings in those locations. Because of some machine displays and challenges in determining product characteristics, we could not collect information on the pricing of some items (508 of 5,815; 8.7%) or portion size (290 of 5,815; 5.0%), although we believe that this absence of data was circumstantial and not systematic. The study was cross-sectional, and thus we could not track changes in availability, prices, and portion sizes over time. We also do not have access to sales or purchase data, which could have clarified purchasing behaviors. Future work should collect data on customers and examine availability and purchases longitudinally.

This research provides novel findings on the availability of food items at highway rest areas in a southern US state using a valid and reliable audit tool and geospatial analysis. We found mostly less-healthful foods being offered, though healthful options were available. Our findings support the need for policy changes to increase the number and presentation of healthful food options at highway rest areas, which could position rest areas as a healthful alternative to less-healthful options for travelers.
